# Correction: HCOOH disproportionation to MeOH promoted by molybdenum PNP complexes

**DOI:** 10.1039/d1sc90239c

**Published:** 2021-11-23

**Authors:** Elisabetta Alberico, Thomas Leischner, Henrik Junge, Anja Kammer, Rui Sang, Jenny Seifert, Wolfgang Baumann, Anke Spannenberg, Kathrin Junge, Matthias Beller

**Affiliations:** Leibniz-Institut für Katalyse e. V. Albert-Einstein Straße 29a 18059 Rostock Germany henrik.junge@catalysis.de matthias.beller@catalysis.de; Istituto di Chimica Biomolecolare, Consiglio Nazionale delle Ricerche tr. La Crucca 3 07100 Sassari Italy elisabetta.alberico@cnr.it

## Abstract

Correction for ‘HCOOH disproportionation to MeOH promoted by molybdenum PNP complexes’ by Elisabetta Alberico *et al.*, *Chem. Sci.*, 2021, **12**, 13101–13119, DOI: 10.1039/D1SC04181A.

The authors regret that in Scheme 2 of the original article, complexes 7 and 8 were drawn incorrectly. The solid-state structure of both complexes, as established by X-ray analysis, had been previously reported (7 (ref. 1) and 8 (ref. 2)). In both complexes, the PNP ligand adopts a facial tridentate coordination to molybdenum and not a meridional one, as erroneously shown in Scheme 2 of the original article. The correct ligand arrangements in the metal coordination sphere for complexes 7 and 8 are reported below in Scheme 1.

**Scheme 1:**
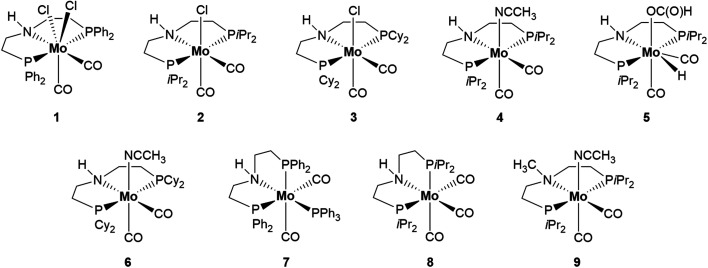
Mo–PNP complexes tested in the dehydrogenation of HCOOH.

Please note that complex 8 is also shown in Scheme 4 in the proposed mechanism for HCOOH decarbonylation (green part), and in Fig. 2. In both cases, the correct structure for complex 8 is reported below in Scheme 2 and Fig. 1.

**Scheme 2:**
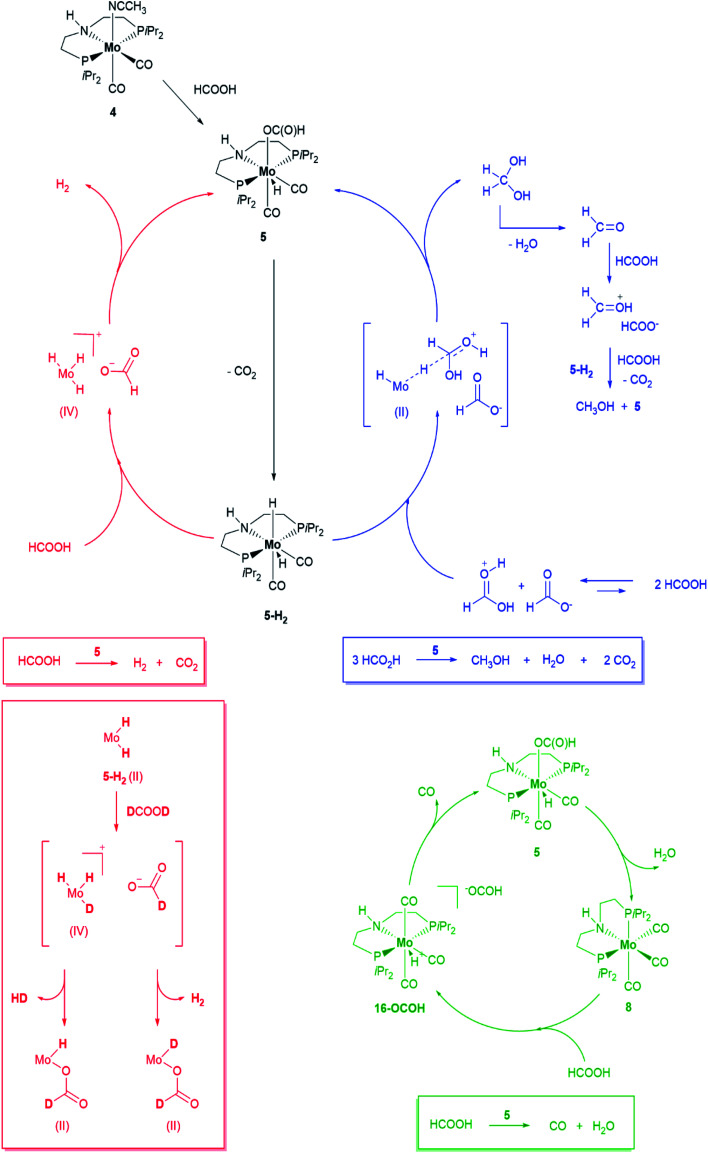
Proposed mechanisms for HCOOH dehydrogenation (red), disproportionation (blue) and decarbonylation (green) promoted by 5. Evidence for the formation of a Mo(iv) species is based on the detection by NMR of H_2_ and HD following addition of DCOOD to Mo(H)_*n*_ species (see Fig. SI-31).

**Fig. 1:**
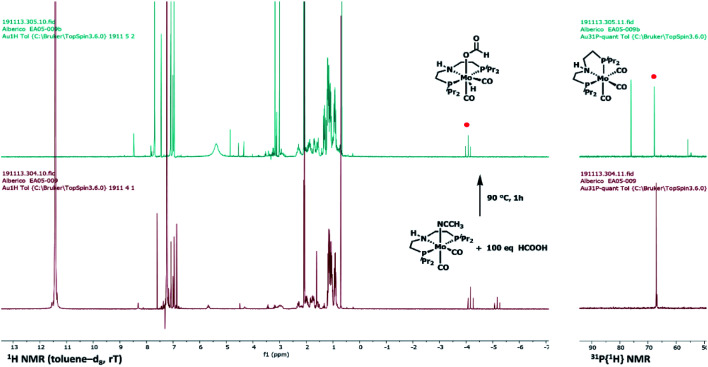
^1^H and ^31^P{^1^H} NMR spectra of a toluene-d_8_ solution of {Mo(CH_3_CN)(CO)_2_(HN[(CH_2_CH_2_P)(CH(CH_3_)_2_)_2_]_2_} 4 in the presence of 100 equivalents of HCOOH ([Mo] 10^−2^ M, [HCOOH] 1 M), before (a) and after heating at 90 °C for 1 hour (b). Spectra were recorded at room temperature. Signals related to complex 5 are marked by red dots.

Furthermore, a mistake was made in the caption of Fig. 6, showing the solid-state structure of complex 9: the latter has been incorrectly described as a Mo(i)-hydride species {Mo(H)(CO)_2_(CH_3_CN)[CH_3_N(CH_2_CH_2_P(CH(CH_3_)_2_)_2_)_2_]}. The correct formula, in agreement with the X-ray structure, is as follows and is shown above in Fig. 2: {Mo(CO)_2_(CH_3_CN)[CH_3_N(CH_2_CH_2_P(CH(CH_3_)_2_)_2_)_2_]}.

**Fig. 2:**
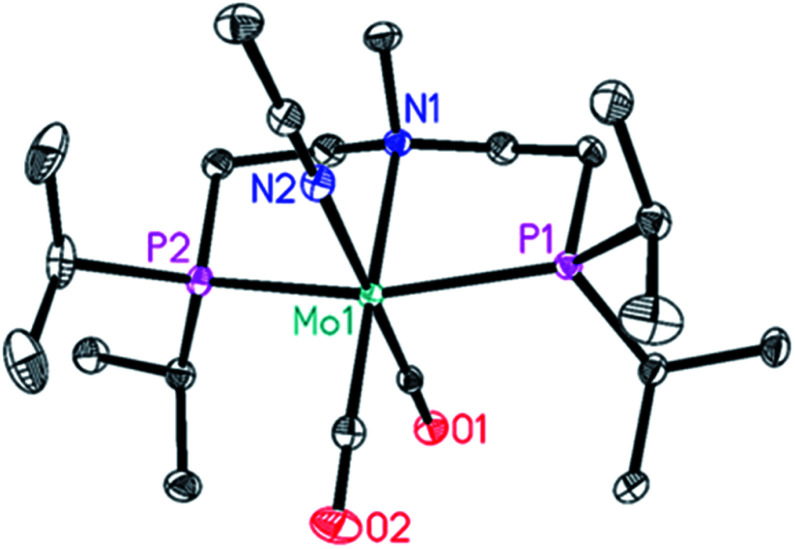
Molecular structure of {Mo(CO)_2_(CH_3_CN)[CH_3_N(CH_2_CH_2_P(CH(CH_3_)_2_)_2_)_2_]} 9. Displacement ellipsoids correspond to 30% probability. Hydrogen atoms are omitted for clarity.

The Royal Society of Chemistry apologises for these errors and any consequent inconvenience to authors and readers.

## Supplementary Material
